# Scavenger guild and consumption patterns of an invasive alien fish species in a Mediterranean wetland

**DOI:** 10.1002/ece3.9133

**Published:** 2022-07-31

**Authors:** Adrian Orihuela‐Torres, Juan Manuel Pérez‐García, José Antonio Sánchez‐Zapata, Francisco Botella, Esther Sebastián‐González

**Affiliations:** ^1^ Department of Applied Biology, Centro de Investigación e Innovación Agroalimentaria y Agroambiental (CIAGRO‐UMH) Miguel Hernández University Orihuela Spain; ^2^ Department of Ecology Alicante University Alicante Spain

**Keywords:** aquatic‐terrestrial interface, aquatic subsidies, carrion, common carp, nutrient cycling

## Abstract

Invasive Alien Species (IAS) alter ecosystems, disrupting ecological processes and driving the loss of ecosystem services. The common carp *Cyprinus carpio* is a hazardous and widespread IAS, becoming the most abundant species in many aquatic ecosystems. This species transforms ecosystems by accumulating biomass to the detriment of other species, thus altering food webs. However, some terrestrial species, such as vertebrate scavengers, may benefit from dead carps, by incorporating part of the carp biomass into the terrestrial environment. This study describes the terrestrial vertebrate scavenger assemblage that benefits from carp carcasses in a Mediterranean wetland. We also evaluate the seasonal differences in the scavenger assemblage composition and carrion consumption patterns. Eighty carp carcasses (20 per season) were placed in El Hondo Natural Park, a seminatural mesohaline wetland in south‐eastern Spain, and we monitored their consumption using camera traps. We recorded 14 scavenger species (10 birds and four mammals) consuming carp carcasses, including globally threatened species. Vertebrates consumed 73% of the carrion biomass and appeared consuming at 82% of the carcasses. Of these carcasses consumed, 75% were completely consumed and the mean consumption time of carcasses completely consumed by vertebrates was 44.4 h (SD = 42.1 h). We recorded differences in species richness, abundance, and assemblage composition among seasons, but we did not find seasonal differences in consumption patterns throughout the year. Our study recorded a rich and efficient terrestrial vertebrate scavenger assemblage benefitting from carp carcasses. We detected a seasonal replacement on the scavenger species, but a maintenance of the ecological function of carrion removal, as the most efficient carrion consumers were present throughout the year. The results highlight the importance of vertebrate scavengers in wetlands, removing possible infectious focus, and moving nutrients between aquatic and terrestrial environments.

## Graphical abstract

The common carp (Cyprinus carpio) is a worldwide hazardous Invasive Alien Species, becoming the most abundant species in many aquatic ecosystems. This study has recorded a rich and efficient vertebrate terrestrial scavenger assemblage in a Mediterranean wetland that benefits from carp carcasses. The results highlight the importance of vertebrate scavengers in wetlands, removing possible infectious focus, and moving nutrients between aquatic and terrestrial environments.

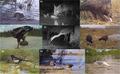

## 1 INTRODUCTION

The introduction of invasive alien species (IAS) is one of the greatest threats to biodiversity, economies, and human health (Bellard et al., 2016; Peyton et al., 2019). IAS alter ecosystems, triggering the disruption of ecological processes and the loss of ecosystem services (Pejchar & Mooney, 2009). These ecological and economic impacts have been studied to a lesser extent in aquatic compared to terrestrial ecosystems (Cuthbert et al., 2021). However, freshwater ecosystems have been specially affected by IAS, jeopardizing the functioning of these highly threatened ecosystems (Simberloff et al., 2013; Strayer, 2010).

One of the most pervasive and destructive IAS to aquatic ecosystems is the common carp *Cyprinus carpio* (hereafter carp; Lowe et al., 2000), which has been introduced to at least 120 countries (Casal, 2006). Throughout most of its range, carp is an invasive species that performs as an ecosystem engineer (Matsuzaki et al., 2009), invading all types of aquatic ecosystems. It feeds by sucking the sediment and filtering it with the mouth, increasing water turbidity, uprooting plants, and predating on benthic species (Zambrano & Hinojosa, 1999). Introduced in Spain in the 17th century, it has spread widely throughout the country, becoming the most abundant species in many lentic aquatic systems (Blanco et al., 2003; Godinho & Ferreira, 1998). In carp‐dominated ecosystems, carp biomass accumulates to the detriment of macrophytes and macroinvertebrates, altering the food web and ecological processes (Dalu et al., 2020; Maceda‐Veiga et al., 2017). The transference of nutrients between the aquatic and terrestrial ecosystems is often also modified, as living carps may subsidize terrestrial predators (Gil‐Sánchez, 1994), and dead carps may reach the shores and thus be exploited by terrestrial scavengers (Dunlop et al., 2020; Schlichting et al., 2019).

The importance of aquatic subsidies to the terrestrial environment has been widely highlighted (Cederholm et al., 1999), affecting the structure and functioning of ecosystems (Polis et al., 1997). These subsidies affect from the base of the food webs such as the soil (Irick et al., 2015), vegetation (Ben‐David et al., 1998), or arthropods (Hoekman et al., 2011) to vertebrates such as reptiles (Lillywhite et al., 2008), birds (Field & Reynolds, 2011), mammals (Roth, 2003), and top predators (Darimont et al., 2008). One of the guilds involved in the aquatic–terrestrial nutrient transport are terrestrial vertebrate scavengers, which can consume a large amount of biomass from aquatic systems and incorporate it into terrestrial ecosystems (Hewson, 1995; Levi et al., 2015). However, most of the studies on nutrient transport by carrion and scavengers at the aquatic–terrestrial interface have been conducted at the coastline (Brown et al., 2015; Huijbers et al., 2013, 2016), on islands (Redondo‐Gómez et al., 2022), and rivers (Schlichting et al., 2019; Shardlow & Hyatt, 2013). Despite being one of the most productive ecosystems on Earth and extremely valuable for humans (Zedler & Kercher, 2005), studies of carrion consumption patterns in wetlands are scarce. To the best of our knowledge, the few studies carried out in wetlands monitored the consumption of bird (Gabel et al., 2019; Hiraldo et al., 1991; Linz et al., 1991; Pain, 1991), amphibian, and reptile carcasses (Abernethy et al., 2017), never covering more than one season.

A large number of species can consume carrion (Sebastián‐González et al., 2019). This has highlighted the critical role scavengers play in ecosystems, being present in almost half of the aquatic and terrestrial trophic links worldwide (Wilson & Wolkovich, 2011). In addition to maintaining and stabilizing food webs (DeVault et al., 2003), vertebrate scavengers perform essential ecological functions by contributing to rapid and effective nutrient recirculation (Wilson & Read, 2003). They also efficiently consume carrion, removing potential disease focuses (Markandya et al., 2008). However, the performance of this ecological process is mediated by an array of factors, one of the most important of which is seasonality (Peers et al., 2020). In cold or dry seasons, when food is scarce, carrion is used by more vertebrates and it is consumed more efficiently (Pereira et al., 2014; Turner et al., 2020). When temperature raises, invertebrates are more active and vertebrates may reduce their consumption (DeVault et al., 2004). This factor might be even more relevant in Mediterranean wetlands, where there are long periods of drought and high temperatures and many wetlands dry out, causing drastic changes in the ecosystem (Melendez‐Pastor et al., 2010).

The objective of this study was to describe the terrestrial vertebrate scavenger assemblage that benefits from alien invasive carp carcasses in a Mediterranean wetland (El Hondo Natural Park), as well as to determine the consumption efficiency of the scavenger assemblage according to season. There is a lack of information on carp carcass availability over space and time, being carrion availability estimation a major challenge in carrion ecology (Moleón et al., 2019). Nevertheless, in our study area there are different sources of carp carcasses linked to random natural deaths (e.g., illness, accidental trapping, predation) which provide an unpredictable number of carcasses and mass mortality events linked to natural (e.g., drought, parasites) or human‐mediated (e.g., fishing, pond desiccation) causes. In any case, carp biomass is very large as revealed by the large amount of carp biomass removed annually from the larger ponds (30,000–50,000 kg/year in 2004–2014, El Hondo Natural Park management team, pers. comm.). Here, we aim to study the scavenger assembly associated to single carcasses as a first step to analyze the role of fish scavenging in wetlands. We expect that the scavenger assemblage and the carrion consumption patterns will change throughout the year, as many scavenger species are migratory and the availability of resources varies during the year, which could have an impact in carrion removal and ultimately in nutrient cycling.

## 2 METHODS

### 2.1 Study area

This study was performed in El Hondo Natural Park (2387 ha; El Hondo, hereafter), in south‐eastern Spain (38°11′16″N, 0°47′28″O). El Hondo is a seminatural mesohaline coastal wetland composed of two large ponds that are used for crop irrigation and about 20 smaller pons that are used for conservation or as waterfowl hunting grounds and sport fishing. Water supplies come mainly from pumping water from the Segura river, but also from agricultural drainage channels and from natural surface runoff. The ponds are separated by channels and dykes. Ponds are shallow and surrounded by reed *Phragmites australis*, the dominant vegetation in the park. There are areas with *Schoenoplectus litoralis* and *Bolboschoenus maritimus* and large extensions of saltmarshes with vegetation typical of this ecosystem, such as *Salicornia* spp., *Halocnemum strobilaceum*, *Limonium* spp., and *Tamarix* spp. The park is surrounded by irrigated agricultural fields and palm crops of *Phoenyx* spp. and *Washingtonia* spp. Climate is dry thermo‐Mediterranean with an average temperature of 18°C and an annual rainfall of less than 300 mm, with most of the precipitation accumulating in autumn and spring.

El Hondo is a Ramsar site protected by the European Habitat and Bird Directive (Directive 92/43/CEE), it is also a ZEPA area and it is included in the NATURA‐2000 network of the European Union. This wetland is an important wintering and breeding area for many waterfowl species, including the globally endangered marbled teal *Marmaronetta angustirostris* and white‐headed duck *Oxyura leucocephala*. It is also habitat of several species of raptors, gulls and medium‐sized carnivores, such as the red fox *Vulpes vulpes*, the common genet *Genetta genetta* and the beech marten *Martes foina*, that are potential scavengers.

### 2.2 Data sampling

**FIGURE 1:**
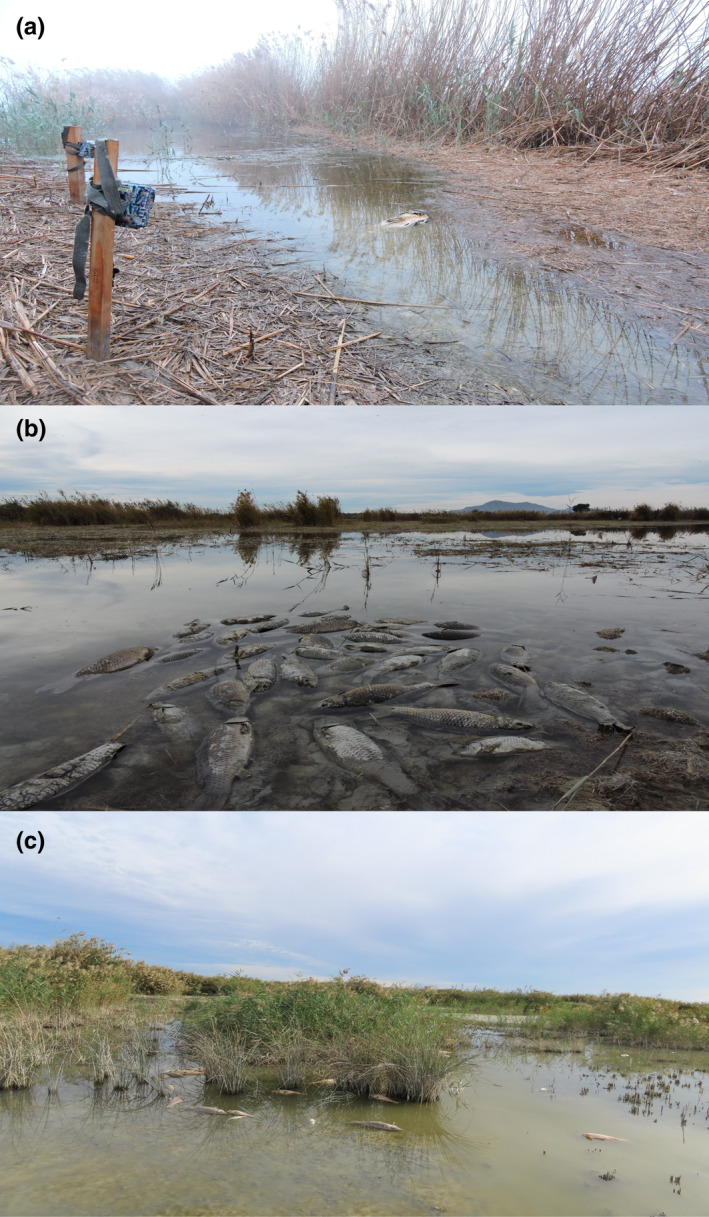
Carcasses of common carp in El Hondo Natural Park, south‐eastern Spain. (a) Shows camera placement with a carcass of common carp mimicking natural mortality on the shore of the pond. (b) and (c) show common carp killed by anoxia in late summer.

Between May 2020 and March 2021, we placed 80 carp carcasses (20 per season: spring, summer, autumn, and winter; mean weight 377 g; range 100–1302 g; SD = 183.4 g; mean length 40 cm), monitored by camera traps (model: Browning Strike Force pro HD). One of the carcasses was carried away by the water current, and thus excluded from the analyses. The carcasses were placed at randomly chosen locations in the pond shores mimicking natural mortality (Figure 1), as is often observed in the study area, and left for up to 15 days or until their total consumption (i.e., only scales and bones left). Carcasses were placed at least 200 m apart. Some carcasses were placed in a nearby site (<25 m), but always with at least 1 month of difference among them (Turner et al., 2020). Two camera traps were placed for each carcass: one on photo mode, which took three photos every 30 s when activated by movement, and the second one on video mode, which took a 20‐s video every minute when activated by movement. The video mode allowed us to confirm consumption in case of doubt. The carps were caught by the Natural Park staff in the park ponds as part of the program for the elimination of invasive species.

### 2.3 Scavenging variables

After reviewing all pictures and videos, we calculated the following variables: (1) “richness”: number of species consuming a carcass. We calculated this variable separately for birds, for mammals and in total; (2) “abundance”: maximum number of different individuals per carcass, consuming simultaneously in a picture, or that can be differentiated by age, sex or marks in different photos or videos (Sebastián‐González et al., 2019). We calculated this variable for birds, for mammals and in total; (3) “detection time”: time needed for a scavenger to find and feed upon the carcass for the first time since it is placed; (4) “consumption time”: time until total carcass consumption since it is placed; (5) “consumed biomass”: percentage of consumed carcass (except scales and bones) at the time the cameras were removed. The percentages were estimated visually at 5% intervals.

### 2.4 Statistical analyses

We fitted Generalized Linear Models (GLMs) in R 3.6.0 (R Core Team, 2019) to test our hypotheses. “Richness” and “abundance” (for mammals, birds, and total), “detection time,” “consumption time,” and “consumed biomass” were the response variables while “season” was the categorical predictor. We also used “richness” and “abundance” as predictors to test their effect on “consumption time” and “consumed biomass.” We used Poisson distribution error for “richness” and “abundance,” except for “bird abundance,” which was fitted using a negative binomial distribution error. We also used Gaussian distribution error for “detection time” and “consumption time,” and binomial error distribution for “consumed biomass.” “Detection time” and “consumption time” were log‐transformed to meet normality. When significant differences appeared (*p* < .05), we used the Tukey test to determine which seasons were different from each other. Furthermore, we used the permutational multivariate analysis of variance (PERMANOVA; Anderson, 2001) to compare the scavenger assemblages composition (species identity and relative abundances) among seasons. For PERMANOVA analyses, we used the *vegan* (Oksanen et al., 2019) package in R.

## 3 RESULTS

### 3.1 Overall scavenger guild and scavenging patterns

**FIGURE 2:**
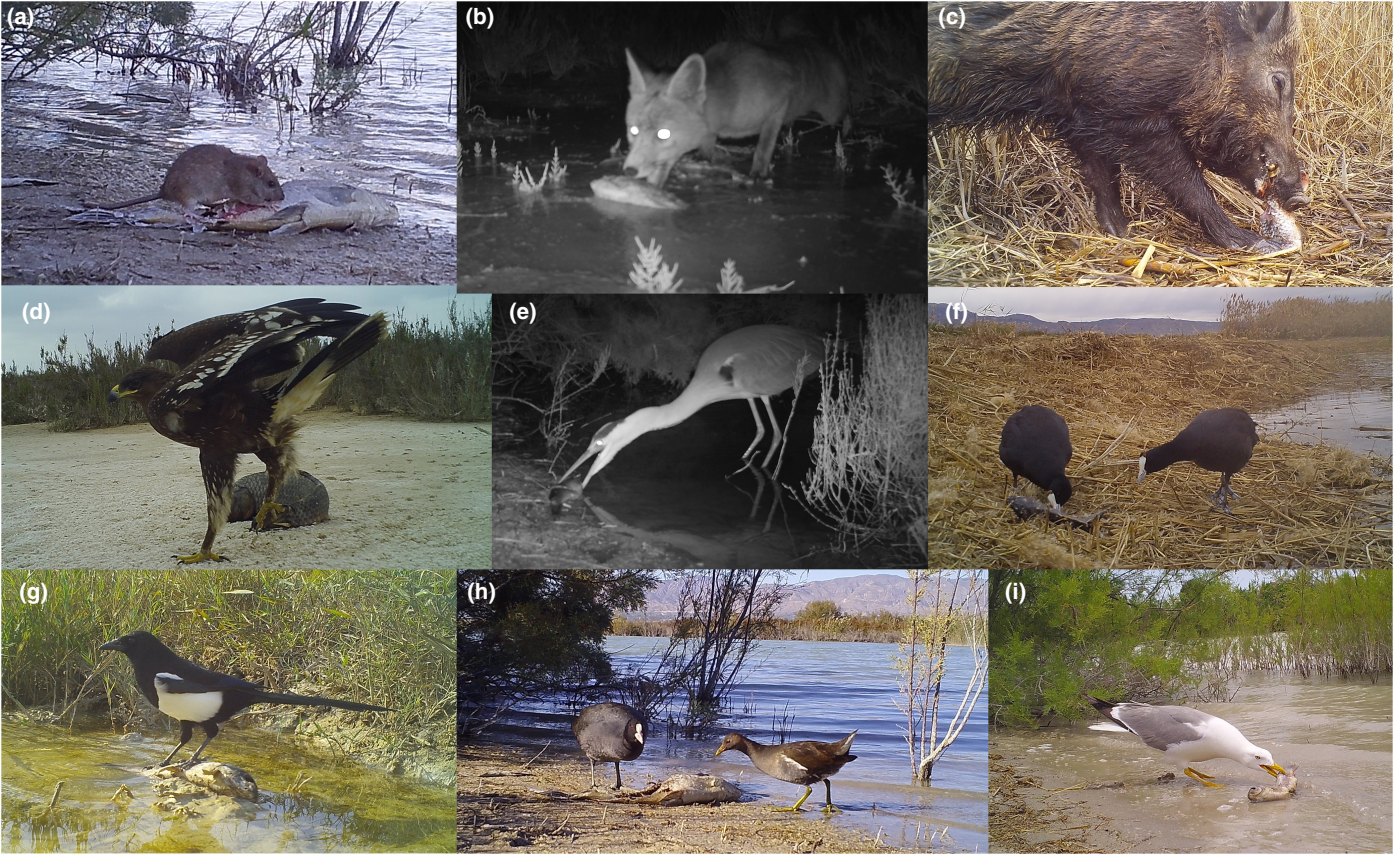
Vertebrate scavenger species recorded in El Hondo Natural Park, south‐eastern Spain, consuming carrion of an invasive alien species, the common carp. The figure shows; (a) brown rat, (b) red fox, (c) wild boar, (d) greater spotted eagle, (e) gray heron, (f) red‐knobbed coot, (g) Eurasian magpie, (h) Eurasian coot and common gallinule, (i) yellow‐legged gull.

We recorded 14 scavenger species (10 birds and four mammals) consuming carp carcasses (Figure 2; Table 1). The most frequent mammal species were the brown rat *Rattus norvegicus*, the red fox, and the wild boar *Sus scrofa*, while among birds were the Eurasian magpie *Pica pica*, the common moorhen *Gallinula chlorophus*, and the black‐headed gull *Chroicocephalus ridibundus*. In addition, we recorded globally endangered species such as the greater spotted eagle *Clanga clanga* and the red‐knobbed coot *Fulica cristata* feeding on carcasses. Vertebrates consumed 73% of the available carrion biomass and were recorded consuming at 82% of the carcasses (*n* = 65). Of these carcasses consumed, 75% (*n* = 49) were completely consumed, 12.5% (*n* = 8) were consumed between 99% and 50%, and 12.5% (*n* = 8) were consumed less than 50%. Twelve carcasses remained untouched and two were completely consumed by invertebrates. The mean consumption time of carcasses completely consumed by vertebrates was 44.4 h (SD = 42.1 h). Mammals detected more carcasses than birds (*n* = 39 vs. 25; 49% vs. 32%, respectively), and were more frequently detected consuming at the carcasses (57%, *n* = 47) compared to birds (39%, *n* = 31). The mean detection time was 27.1 h (SD = 39.9 h). In addition, we found that the percentage of consumed biomass but not consumption time, significantly increased in carcasses consumed by a larger number of species and individuals (Table 2).

**TABLE 1:** Number of common carp carcasses per season and in total in which the different species of vertebrate scavengers consumed in El Hondo Natural Park, south‐eastern Spain.

	Spring	Summer	Autumn	Winter	Total
Red fox *Vulpes Vulpes*	4	1	2	2	9
Wild boar *Sus scrofa*	1	4	1	3	9
Brown rat *Rattus norvegicus*	5	5	10	10	30
Wood mouse *Apodemus sylvaticus*	1	0	0	1	2
Black‐headed gull *Chroicocephalus ridibundus*	6	1	0	0	7
Slender‐billed gull *Chroicocephalus genei*	2	1	0	0	3
Yellow‐legged gull *Larus michahellis*	5	0	0	0	5
Gray heron *Ardea cinerea*	3	1	0	1	5
Common moorhen *Gallinula chlorophus*	2	0	5	1	8
Eurasian magpie *Pica pica*	2	0	5	3	10
Water rail *Rallus aquaticus*	0	0	1	0	1
Eurasian coot *Fulica atra*	0	0	1	0	1
Red‐knobbed coot *Fulica cristata*	0	0	1	0	1
Greater spotted eagle *Clanga clanga*	0	0	2	1	3

**TABLE 2:** Results of the GLMs testing differences among seasons in species richness (total, mammal, and bird richness), abundance (total, mammal, and bird abundance), percentage of carcass consumed (consumed biomass), detection time, and consumption time of the vertebrate scavenger assemblage in El Hondo Natural Park, south‐eastern Spain.

	df	Deviance	Resid. df	Resid. Dev.	Pr(>Chi)
Total richness ~ season	3	8.208	75	53.478	**0.042**
Mammal richness ~ season	3	1.199	75	54.528	0.753
Bird richness ~ season	3	17.350	75	74.383	**0.001**
Total abundance ~ season	3	15.254	75	87.091	**0.002**
Mammal abundance ~ season	3	1.837	75	59.708	0.606
Bird abundance ~ season	3	15.669	75	66.709	**0.001**
Consumed biomass ~ season	3	7.508	75	68.292	0.057
Detection time ~ season	3	7.892	54	85.626	0.173
Consumption time ~ season	3	2.983	36	45.437	0.500
Consumption time ~ total richness time + season	1	0.654	38	16.432	0.418
Consumed biomass ~ total richness + season	1	7.925	77	53.761	**0.005**
Consumption time ~ total abundance + season	1	0.770	38	36.203	0.380
Consumed biomass ~ total abundance + season	1	10.098	77	92.246	**0.001**

*Note*: Moreover, we tested the relationship between consumption time and consumed biomass in relation to abundance and richness of vertebrate scavengers. We show degrees of freedom (df), deviance, residual degrees of freedom (Resid. df), residual deviance (Resid. Dev.) and the *p‐*value. Significant *p*‐values are in bold.

### 3.2 Effect of seasonality

**FIGURE 3:**
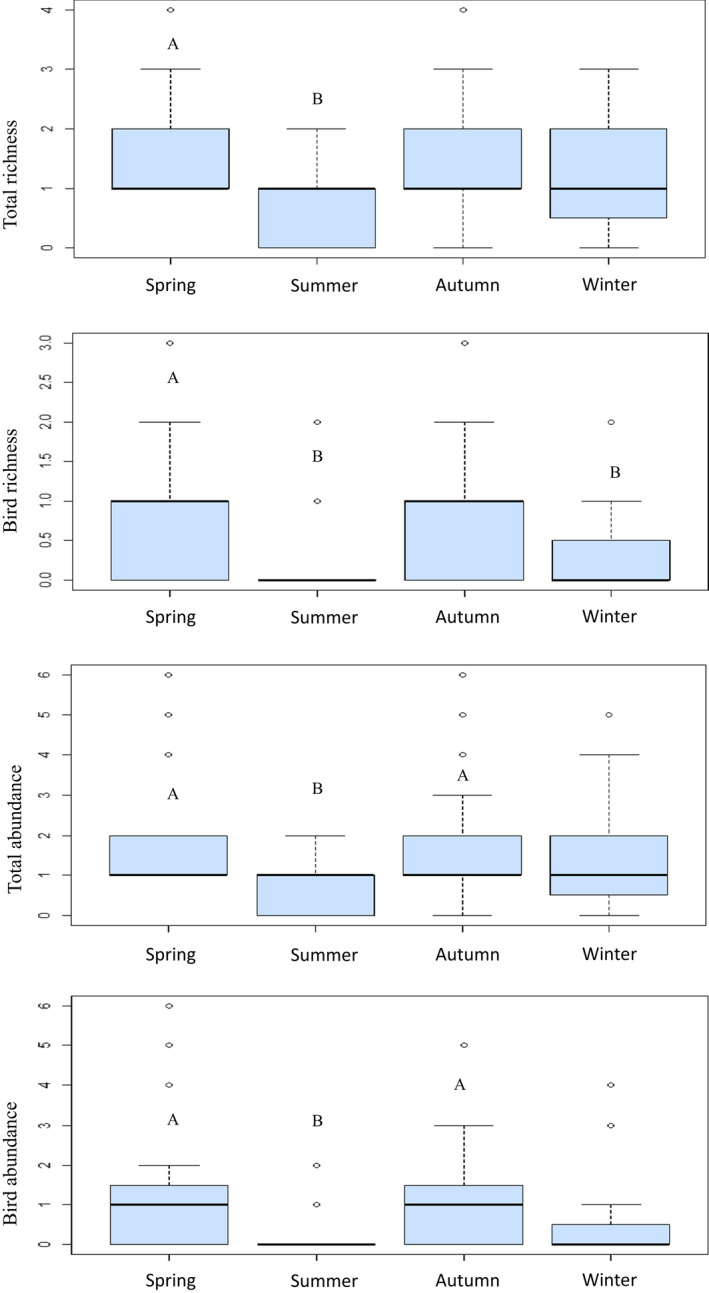
Seasonal changes in the vertebrate scavenger assemblage of common carp carcasses at El Hondo Natural Park, south‐eastern Spain. Figures are boxplots for the variables that showed significant differences between seasons. Letters identify seasons showing statistically significant differences (Tukey test, *p* < .05). See Appendix S1 Table S1 for analysis's details.

**FIGURE 4:**
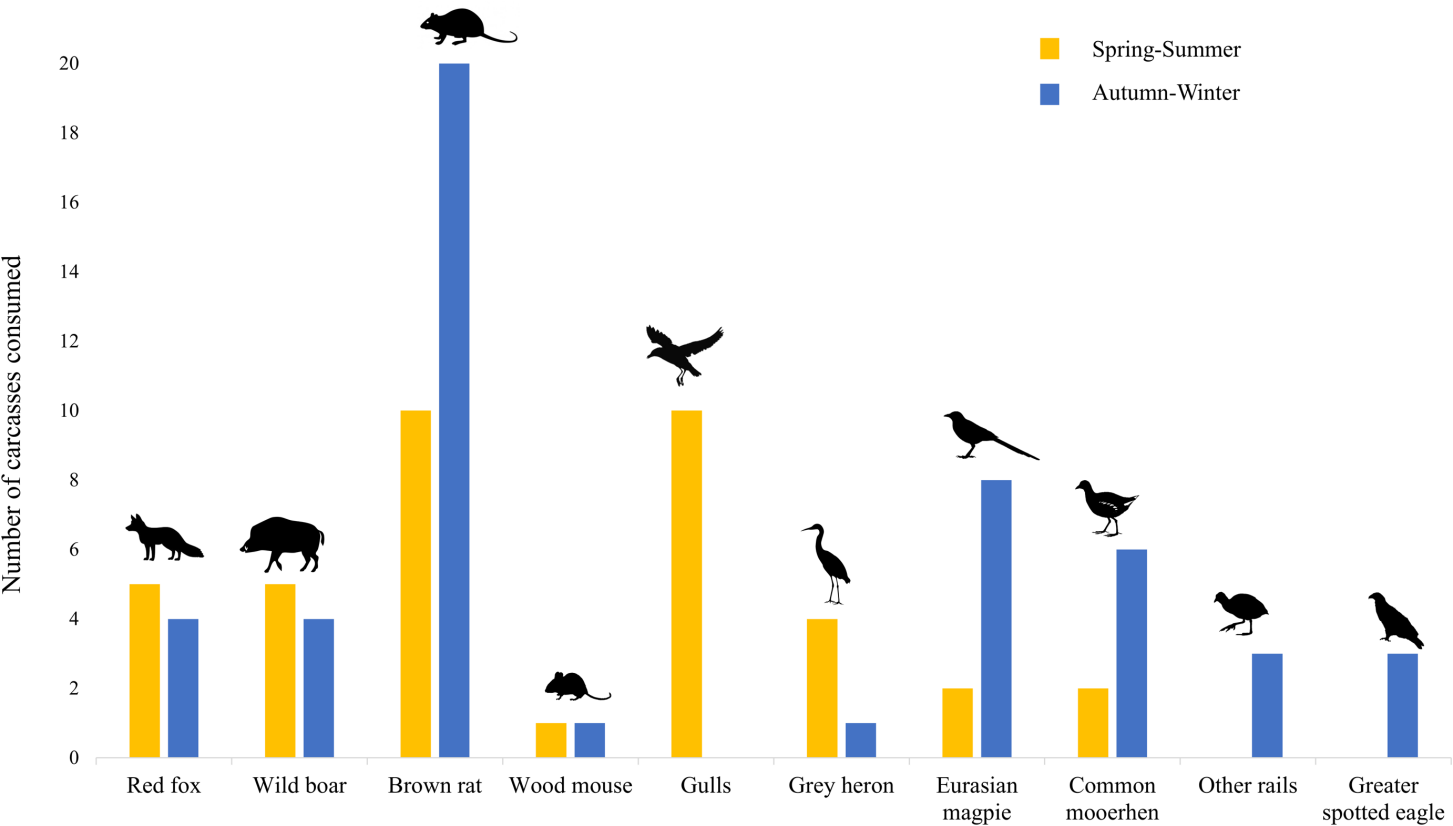
Number of common carp carcasses, by season, in which the different species of vertebrate scavengers were recorded in El Hondo Natural Park, south‐eastern Spain. The gull group consists of the black‐headed gull, the slender‐billed gull and the yellow‐legged gull, and the other rails group is composed by the Eurasian coot, the red‐knobbed coot and the water rail.

We detected sharp seasonal differences in species richness, abundance, and composition of the scavenger assemblage (Figure 3; Appendix S1 Tables S1 and S2). Spring was the season with the highest number of scavenger species (*n* = 10) and when vertebrates appeared in more carcasses (*n* = 20, 100%). In contrast, in summer, we only found six vertebrate scavenger species consuming at 12 (60%) carcasses (Table 3). We found significant differences between spring and summer in total species richness, bird species richness, total abundance, and bird abundance. We also found differences between spring and winter in bird species richness, and between summer and autumn in total abundance and bird abundance (Table 3; Appendix S1 Table S1). However, we found no significant differences in mammal species richness or abundance throughout the year. Regarding the composition of the scavenger assemblage, we found significant differences between spring–summer, spring–autumn, spring–winter, and summer–autumn (Appendix S1 Table S2). Birds were more important in carrion consumption in spring, while mammals were more frequent and were recorded at a greater number of carcasses the rest of the year (Table 3). Three gull species (black‐headed gull, yellow‐legged gull *Larus michahellis*, and slender‐billed gull *Chroicocephalus genei*) and the gray heron *Ardea cinerea* played a key role in spring–summer, while they were absent in autumn–winter, except for the gray heron at one carcass (Figure 4; Table 1). In contrast, rats, magpies, rails, and raptors (i.e., greater spotted eagle) were more frequent and visited more carcasses in autumn–winter (Figure 4). We also detected some uncommon scavenger species, such as coots *Fulica cristata* and *F. atra* and the water rail *Rallus aquaticus*, scavenging in autumn–winter (Figure 4). The most efficient scavengers (i.e., species consuming more carrion in less time) such as red fox and wild boar, were present throughout the year.

**TABLE 3:** Scavenger assemblage and scavenging efficiency of common carp carcasses at El Hondo Natural Park, south‐eastern Spain, in different seasons.

	Winter	Autumn	Spring	Summer	Total
Total richness	1.1	1.4	1.6	0.7	1.2 ± 0.9 (0–4)
Mammal richness	0.8	0.6	0.6	0.5	0.6 ± 0.6 (0–2)
Bird richness	0.3	0.7	1	0.1	0.6 ± 1.3 (0–6)
Total abundance	1.3	1.7	2	0.7	1.5 ± 1.5 (0–6)
Mammal abundance	0.8	0.7	0.6	0.5	0.7 ± 0.6 (0–3)
Bird abundance	0.5	1	1.4	0.2	0.8 ± 1.3 (0–6)
Consumed biomass (%)	83.9	70.5	86.2	51.3	73.3 ± 41.2 (0–100)
Detection time (h)	47.1	15.3	23	23.5	27.1 ± 36.1 (0.9–287.7)
Consumption time (h)	56.1	47.6	32.9	22.6	44.4 ± 37.1 (2.8–187.1)
Species number	8	9	10	6	14
Vertebrate presence	16	17	20	12	65
Bird presence	5	11	13	2	31
Mammal presence	14	13	11	9	47
100% consumed carcasses	15	13	13	8	49

*Note*: We show species richness (for birds, mammals, and total), abundance (for birds, mammals, and total), consumed biomass (the percentage of carrion biomass consumed), detection time, consumption time, species number, vertebrate, bird, and mammal presence (number of carcasses consumed by vertebrates, birds, or mammals, respectively) and 100% consumed carcasses (number of carcasses completely consumed). The table shows mean values, except for the *Total* column, which also shows standard deviation and range for several variables.

We did not find marked seasonal differences in terms of consumption patterns. The average percentage of consumed biomass was higher in spring than in summer (86% vs. 51%, *p*‐value = .057), but remained constant throughout the year (Table 3). In addition, we found no significant differences in detection and consumption times between seasons (Table 2).

## 4 DISCUSSION

Our study has recorded a rich and efficient vertebrate terrestrial scavenger assemblage in a Mediterranean wetland that benefits from carp carcasses. This highlights the large aquatic–terrestrial biomass transfer from an IAS and the importance of an abundant IAS for the terrestrial vertebrate assemblage and the food web in this wetland. The scavenger assemblage included more bird species, although mammals were recorded in more carcasses. Vertebrates completely consumed more than half of the carcasses in less than 2 days, highlighting their important role in efficiently removing carrion. We also detected a seasonal replacement of the scavenger species, but a maintenance of the ecological function of carrion removal, as the most efficient carrion removal species, such as the wild boar and the red fox, were detected throughout the year.

Terrestrial vertebrate scavengers performed an efficient use of an aquatic resource (carp carrion) that is partially transferred to the terrestrial environment. We found that 73% of carp carrion biomass is consumed by vertebrate scavengers, which implies that a large amount of aquatic biomass is immediately incorporated into the terrestrial ecosystem. This consumption of aquatic subsidies by terrestrial scavengers may have a direct effect on carp‐carrion consumers' populations (Roth, 2003; Rose & Polis, 1998), but also on the entire ecosystem (Cederholm et al., 1999). When a resource is very abundant, it can impact local processes such as predation and facilitation, creating more inter‐species links (Escobar‐Lasso et al., 2016), and modifying the population density of some species (Rose & Polis, 1998). Besides, some mammals such as foxes can redistribute aquatic nutrients heterogeneously throughout the terrestrial ecosystem, catching large quantities of carrion for later consumption, or by means of excrements or urine (Ben‐David et al., 1998). However, birds can transport nutrients much farther away (Payne & Moore, 2006), such as gulls that feed in El Hondo but have their breeding areas in other Natural Parks, creating a flow of nutrients between ecosystems.

Our study shows that scavenger birds play a more important role in spring and autumn, consuming less carrion in winter and being almost absent in summer. Gulls breed in large numbers in our study area especially black‐headed gulls, which are scarce in winter. The park is an important wintering area for raptors that are almost absent in spring and summer. This turnover in the bird assemblage was also reflected in carrion consumption. In our study, gulls made up an important part of the scavenger assemblage in spring, while the only raptor scavenger, the greater spotted eagle, did so in autumn–winter. Interestingly, locally abundant raptor species, such as the Eurasian marsh harrier *Circus aeruginosus*, the common buzzard *Buteo buteo*, the booted eagle *Hieraaetus pennatus*, or the common kestrel *Falco tinnunculus*, were not detected in this study, while the scarcest and endangered greater spotted eagle consumed many carp carcasses, indicating the importance of this exotic resource for the only wintering area of this species in Spain (Pérez‐García et al., 2020).

Another noteworthy result is the large carrion consumption by the common moorhen, mostly in autumn–winter. This is an omnivorous species with fairly unknown scavenging habits, except for occasional consumption of fish carcasses (Hewson, 1995). The rails that scavenged sporadically were also recorded in autumn–winter, when other food sources are scarcer. Although this is a rare behavior in rails, there are records of scavenging in closely related species, such as the American coot *Fulica americana* (Paullin, 1987), the gray‐cowled wood‐rail *Aramides cajaneus* (Silva e Silva & Olmos, 2016) and even of the red‐knobbed coot (Taylor, 2020), so it may be a more common behavior than previously thought in this waterbird group. The increase in carrion consumption in autumn–winter was also recorded in other scavenging species such as brown rats and Eurasian magpies, that consumed much more carrion at this time of the year when food is scarce, while in spring–summer they may prefer insects, eggs, and nestlings (Pereira et al., 2014). Moreover, we found that larger scavenger species, such as the red fox and the wild boar consumed all carcasses completely, while smaller species such as the brown rat left a large percentage of carcasses partially consumed and were shared with other scavenger species. A similar pattern was found in Belarus, where rodents and magpies appeared in more carp carcasses, but medium‐sized mammals consumed more carrion (Schlichting et al., 2019).

Despite El Hondo being a human‐managed area, we recorded a rich scavenger assemblage with 14 scavenger species. Other studies using fish carcasses in the Australian coastline (Brown et al., 2015; Huijbers et al., 2013, 2016; Schlacher et al., 2013), at rivers in Canada (Shardlow & Hyatt, 2013), Alaska (Levi et al., 2015), or Norway (Dunlop et al., 2020), and on an island in Spain (Redondo‐Gómez et al., 2022), ranged between one and 11 scavenger species. The only studies on scavenger assemblages with fish carcasses that recorded more species were held in the Chernobyl Exclusion Zone, Belarus, a much better preserved area, where 15 scavengers were found (Schlichting et al., 2019), and on the Olympic Peninsula, Washington, where 22 scavenger species were recorded (Cederholm et al., 1989), but they used a much larger sample size (*n* = 945 carcasses). Other studies conducted in wetlands with amphibian and reptile carcasses in USA (Abernethy et al., 2017) and with goose carcasses in southern Spain (Hiraldo et al., 1991) also recorded a lower number of scavenging species (*n* = 7 and 13 species, respectively). In addition, several studies have used IAS carcasses. Dunlop et al. (2020) used Pacific pink salmon *Oncorhynchus gorbuscha* carcasses at rivers in Norway, where they found that, in addition to several species of corvids and gulls, the red fox was the species that consumed the most carrion, similar to our study. On the other hand, in Hawaii, Abernethy et al. (2016) used different IAS carcasses and recorded that all the vertebrate scavengers that consumed them were invasive species, which feeds back into the IAS problem on islands. In our study, the brown rat IAS was recorded in a large number of carcasses, which suggests that the introduction of IAS into the ecosystem benefit other IAS species.

The species‐rich assemblage observed in our study may be one of the causes of the high percentage of consumed biomass, as we found that species richness positively influenced this consumption. In addition, the scavenger assemblage in our study could be richer (see Appendix S1 Figure S1) since several species such as the Eurasian otter *Lutra lutra* and the viperine snake *Natrix maura* approached the carcasses although they did not consume them, even though we know that they are consumers of fish carrion (Cederholm et al., 1989; Hewson, 1995; pers. obs.). This may be due to the large availability of live carp in the Natural Park. Furthermore, although there were no significant differences in carrion consumption patterns throughout the year, in summer, scavengers completely consumed less carcasses and less biomass, which may be related to the availability of carp carrion in the Natural Park, as this is when water level is the lowest and ponds dry out, resulting in the highest annual carp mortality.

Carps have become the most abundant and biomass‐rich species in many wetlands worldwide (Stuart et al., 2021), as is the case in our study area. The large amount of biomass as well as the invasive potential of this species causes a great impact on the ecosystems (Maceda‐Veiga et al., 2017). This study shed light on how this alien species can alter, not only aquatic environments by increasing turbidity, nutrient levels and phytoplankton and decreasing benthic invertebrates, macrophytes (Matsuzaki et al., 2009) and diving ducks' populations (Maceda‐Veiga et al., 2017), but also the surrounding terrestrial ecosystems and its wildlife by changing the nutrient transfer at the aquatic‐terrestrial interface. Our study shows, for the first time, how invasive alien common carps can play a major role on vertebrate scavenger populations in wetlands.

We found a larger importance of an IAS carrion for many scavenger species than previously thought, including globally threatened species. These findings underline the importance of an IAS in the functioning of the food web in a critical and threatened ecosystem. The results shed light on the importance of scavengers in wetlands, removing possible infectious focus for bacteria such as *Clostridium botulinum*, which has caused large mortality events in Mediterranean wetlands (Vidal et al., 2013), as well as to move nutrients between aquatic and terrestrial environments (Cederholm et al., 1999). Future research on the impact of nutrient transport from IAS among ecosystems by vertebrate scavengers is critical to understand the role of scavengers in nutrient cycling and in maintaining this ecological process in one of the most threatened ecosystems on the planet.

## AUTHOR CONTRIBUTIONS

**Adrian Orihuela‐Torres:** Conceptualization (equal); data curation (equal); formal analysis (equal); investigation (equal); methodology (equal); validation (equal); writing – original draft (equal); writing – review and editing (equal). **Juan Manuel Pérez‐García:** Conceptualization (equal); supervision (equal); validation (equal); writing – review and editing (equal). **José Antonio Sánchez‐Zapata:** Conceptualization (equal); methodology (equal); supervision (equal); writing – review and editing (equal). **Francisco Botella:** Conceptualization (equal); investigation (equal); methodology (equal); supervision (equal); writing – review and editing (equal). **Esther Sebastián‐González:** Conceptualization (equal); formal analysis (equal); funding acquisition (equal); methodology (equal); supervision (equal); writing – original draft (equal); writing – review and editing (equal).

## CONFLICT OF INTEREST

The authors declare that they have no conflict of interest.

## Supporting information


Appendix S1

## Data Availability

The dataset is provided in Orihuela‐Torres et al. (2021). All data used in this study can be found at Figshare https://doi.org/10.6084/m9.figshare.16722589 (Orihuela‐Torres et al., 2021).
