# Supplementary material for: Scavenger guild and consumption patterns of an invasive alien fish species in a Mediterranean wetland
Source: Ecol Evol. 2022 Jul 31;12(8):e9133. doi: 10.1002/ece3.9133 (PMC9339756; doi:10.1002/ece3.9133)

# Appendix

**Table S1.** Post-hoc analyses for those models in Table S2 that found significant differences among seasons, including species richness (total and bird richness) and abundance (total and bird abundance) of the vertebrate scavenger assemblage. We show the estimate and the standard error (SE) of the univariate GLMs and the *p*-value. Significant *p-*values are in bold.

|  | Estimate | SE | *p*_value |
| --- | --- | --- | --- |
| Total richness~season |  |  |  |
| Autumn-Winter | 0.241 | 0.285 | 0.830 |
| Spring-Winter | 0.375 | 0.277 | 0.526 |
| Summer-Winter | -0.475 | 0.350 | 0.526 |
| Spring-Autumn | 0.133 | 0.259 | 0.955 |
| Summer-Autumn | -0.716 | 0.336 | 0.140 |
| Summer-Spring | -0.849 | 0.329 | **0.047** |
| Bird richness~season |  |  |  |
| Autumn-Winter | 0.916 | 0.483 | 0.218 |
| Spring-Winter | 1.204 | 0.465 | **0.045** |
| Summer-Winter | -0.642 | 0.707 | 0.792 |
| Spring-Autumn | 0.288 | 0.342 | 0.827 |
| Summer-Autumn | -1.558 | 0.632 | 0.061 |
| Summer-Spring | -1.846 | 0.619 | **0.014** |
| Total abundance~season |  |  |  |
| Autumn-Winter | 0.259 | 0.256 | 0.738 |
| Spring-Winter | 0.418 | 0.248 | 0.326 |
| Summer-Winter | -0.680 | 0.338 | 0.179 |
| Spring-Autumn | 0.158 | 0.230 | 0.900 |
| Summer-Autumn | -0.939 | 0.325 | **0.019** |
| Summer-Spring | -1.097 | 0.318 | **0.003** |
| Bird abundance~season |  |  |  |
| Autumn-Winter | 0.693 | 0.505 | 0.508 |
| Spring-Winter | 1.065 | 0.489 | 0.125 |
| Summer-Winter | -1.153 | 0.736 | 0.389 |
| Spring-Autumn | 0.371 | 0.435 | 0.824 |
| Summer-Autumn | -1.846 | 0.701 | **0.040** |
| Summer-Spring | -2.217 | 0.690 | **0.007** |

**Table S2:** Comparison of vertebrate scavenger assemblages of the El Hondo Natural Park, south-eastern Spain, among seasons by means of permutational multivariate analysis of variance (PERMANOVA). We show the seasons (compared seasons), Df (degrees of freedom), SS (sum of squares), R^2^ (pseudo R^2^), F (pseudo F-statistic) and the *p-*value. Significant *p*-values are in bold.

| **Seasons** | **Df** | **SS** | **R^2^** | **F** | ***p-*value** |
| --- | --- | --- | --- | --- | --- |
| All seasons | 3 | 8.127 | 0.085 | 2.300 | **0.001** |
| Residual | 74 | 87.152 | 0.915 |  |  |
| Total | 77 | 95.278 | 1 |  |  |
|  |  |  |  |  |  |
| Spring-Summer | 1 | 3.011 | 0.067 | 2.575 | **0.010** |
| Residual | 36 | 42.095 | 0.933 |  |  |
| Total | 37 | 45.106 | 1 |  |  |
|  |  |  |  |  |  |
| Summer-Autumn | 1 | 2.939 | 0.076 | 3.037 | **0.009** |
| Residual | 37 | 35.811 | 0.924 |  |  |
| Total | 38 | 38.751 | 1 |  |  |
|  |  |  |  |  |  |
| Autumn-Winter | 1 | 0.750 | 0.016 | 0.632 | 0.693 |
| Residual | 38 | 45.057 | 0.984 |  |  |
| Total | 39 | 45.807 | 1 |  |  |
|  |  |  |  |  |  |
| Spring-Autumn | 1 | 4.566 | 0.075 | 2.983 | **0.003** |
| Residual | 37 | 56.643 | 0.925 |  |  |
| Total | 38 | 61.209 | 1 |  |  |
|  |  |  |  |  |  |
| Spring-Winter | 1 | 3.800 | 0.069 | 2.739 | **0.004** |
| Residual | 37 | 51.340 | 0.931 |  |  |
| Total | 38 | 55.140 | 1 |  |  |
|  |  |  |  |  |  |
| Summer-Winter | 1 | 1.245 | 0.039 | 1.510 | 0.155 |
| Residual | 37 | 30.509 | 0.960 |  |  |
| Total | 38 | 31.754 | 1 |  |  |

**Figure S1**. Species accumulation curve of the vertebrate scavenger assemblage of the El Hondo Natural Park, south-eastern Spain. The X axis shows the number of carcasses placed and the Y axis the number of vertebrate scavenger species that consumed them.


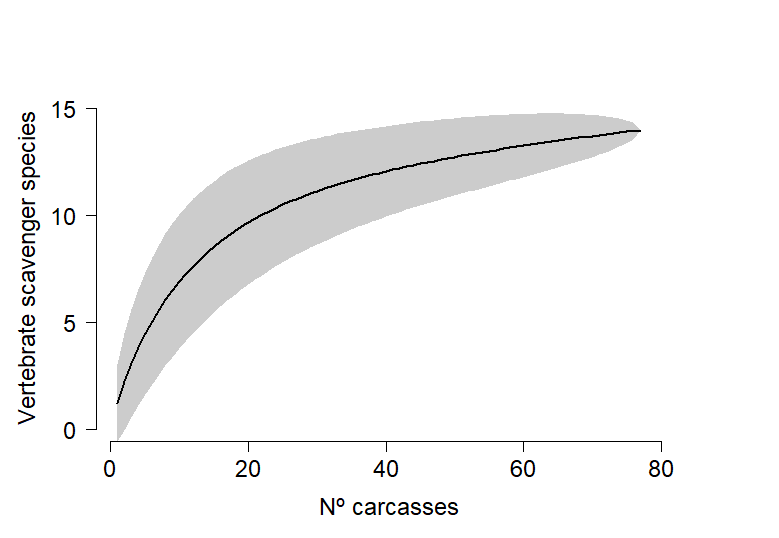

Supplement: Supplementary file 1 — Appendix S1 [file ECE3-12-e9133-s001.docx]
